# Short uncemented stems allow greater femoral flexibility and may reduce peri-prosthetic fracture risk: a dry bone and cadaveric study

**DOI:** 10.1007/s10195-015-0343-1

**Published:** 2015-02-21

**Authors:** Christopher Jones, Adeel Aqil, Susannah Clarke, Justin P. Cobb

**Affiliations:** MSK Lab, 7th Floor, Lab Block, Department of Surgery and Cancer, Imperial College London, Charing Cross Hospital, Fulham Palace Road, London, W6 8RF UK

**Keywords:** Short stem, Total hip arthroplasty, Mechanical testing, Fracture

## Abstract

**Background:**

Short femoral stems for uncemented total hip arthroplasty have been introduced as a safe alternative to traditional longer stem designs. However, there has been little biomechanical examination of the effects of stem length on complications of surgery. This study aims to examine the effect of femoral stem length on torsional resistance to peri-prosthetic fracture.

**Materials and methods:**

We tested 16 synthetic and two paired cadaveric femora. Specimens were implanted and then rapidly rotated until fracture to simulate internal rotation on a planted foot, as might occur during stumbling. 3D planning software and custom-printed 3D cutting guides were used to enhance the accuracy and consistency of our stem insertion technique.

**Results:**

Synthetic femora implanted with short stems fractured at a significantly higher torque (27.1 vs. 24.2 Nm, *p* = 0.03) and angle (30.3° vs. 22.3°, *p* = 0.002) than those implanted with long stems. Fracture patterns of the two groups were different, but showed remarkable consistency within each group. These characteristic fracture patterns were closely replicated in the pair of cadaveric femora.

**Conclusions:**

This new short-stemmed press-fit femoral component allows more femoral flexibility and confers a higher resistance to peri-prosthetic fracture from torsional forces than long stems.

## Introduction

Short femoral stems for uncemented total hip arthroplasty (THA) have been introduced widely, with the suggestion that they may facilitate easier revision [1], distribute stress anatomically [2] and cause fewer intra-operative complications than longer stem designs [3]. With some series reporting 10–16-year survival rates of 99–100 % [4, 5], short stems may be considered a safe alternative to traditional longer stem designs. However, there has been little biomechanical examination of the effects of stem length on complications of surgery.

Peri-prosthetic fracture following primary THA is estimated to occur in approximately 1 % of cases, rising to 4 % within 5 years for revision cases where longer stems are used [6, 7]. Fracture is associated with increased morbidity and dysfunction [8, 9]. Previous studies in cemented stems have found that short stems do not confer a higher risk of peri-prosthetic fracture [10]. The majority of stems inserted worldwide are uncemented and little has been published about the effect of stem length on peri-prosthetic fracture pattern in these press-fit stems. The fracture pattern is also relevant, as it determines treatment and may affect subsequent morbidity [11, 12].

The aim of this study was to examine the impact of femoral stem length on (1) the resistance to fracture of implanted stems subjected to torsional forces, and (2) the peri-prosthetic fracture patterns in a synthetic bone model. Finally, we wished to assess the clinical relevance of this model by comparing tested synthetic femurs to results obtained by testing a single pair of cadaveric bones.

## Materials and methods

In order to compare the two femoral prostheses, we implanted them into synthetic (and later paired cadaveric) femurs. These femurs were then subjected to torsional mechanical testing.

**Fig. 1:**
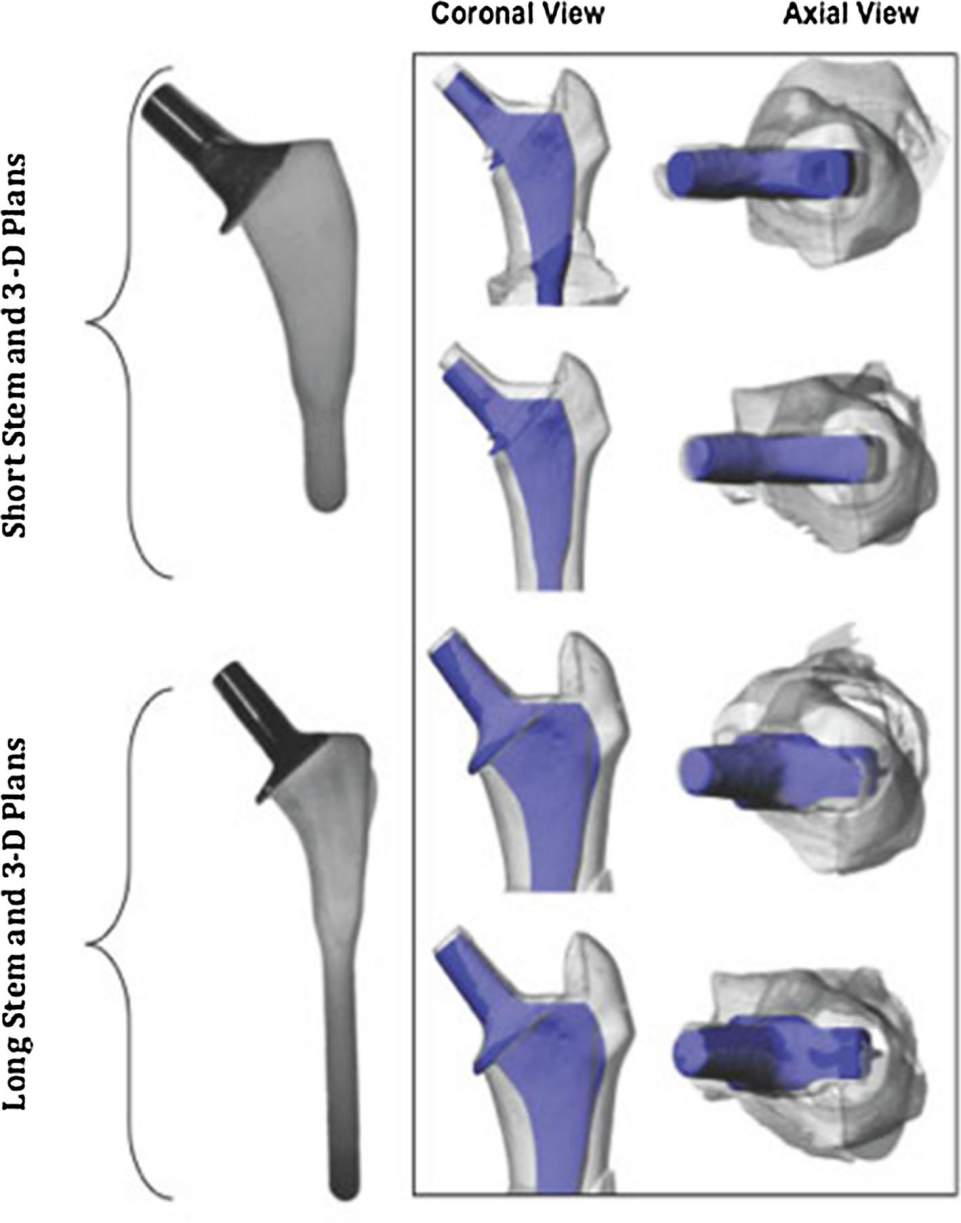
Photograph of the short- and long-stemmedprostheses with three-dimensional rendered images of implanted femurs (shown in *grey*) and the planned positioning of the implant (shown in *blue*) (colour figure online)

This study compared a successful uncemented long-stem design with a shorter one. From shoulder to tip, the longer stems measured 152 mm, while the shorter ones were 100 mm. Besides the apparent difference in length, the shorter stem had a wider proximal section, and was reduced laterally to make insertion easier and minimise the risk of fracture of the greater trochanter. Both stems were fully hydroxyapatite-coated with 12/14 neck tapers and collars to prevent implantation past the required depth (Fig. 1). These stems also required different femoral preparations. The short-stem rasps were designed to be more bone-sparing by impacting loose bone, while the longer stem rasp was designed for more bone extraction.

**Fig. 2:**
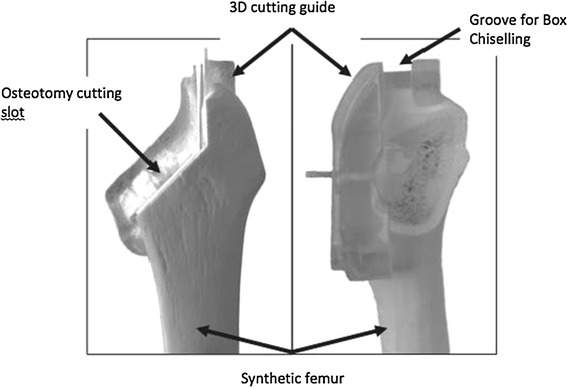
Photograph of a synthetic femur in antero-posterior and lateral plane, following osteotomy of femoral neck using the three-dimensional cutting guide (Embody, UK)

Left-sided “medium”-sized synthetic composite femoral bones (Sawbone Model Number 1121; Sawbones Europe AB, Sweden) were used for their consistency of geometry and to aid a repeatable and controllable methodology (Fig. 2). These bones were dual density, with a foam polyurethane cortical shell. Bones from the same batch were used to avoid any inter-batch variation in mechanical properties. One synthetic bone was scanned by computed tomography (CT) to generate a digital three-dimensional (3D) model, which was later used for planning and validation of correct implant positioning.

A 3D surgical plan was made by one of the authors (S.C.) using the CT scan data from the synthetic bone, and 3D data files of the implants. Ideal positioning for each implant was determined based on alignment of the implant neck and head within the original bone (Fig. 1). From this data, the optimal position for the neck osteotomy and box chiselling entry point could be determined and planned.

Two 3D cutting guides (Embody, UK)—one for each femoral stem—were produced to ensure accuracy and repeatability of our osteotomy cuts and our box chiselling. These guides are designed to precisely match the surface anatomy of the bone (Fig. 2).

Use of these guides ensured that cutting and box chiselling of bone was restricted to areas pre-defined by the 3D planning. Subsequent reaming and rasping thus began in the correct location and planes.

We began by pinning the cutting guide to the specimen. The specimen-matched guide then directed the neck osteotomy and box chiselling of the femoral shaft (Fig. 2). Each bone was sequentially reamed and rasped according to the manufacturer’s instructions.

An experienced surgeon (J.P.C.) used standard intra-operative techniques to determine the appropriate implant size. A size 11 was used for the long, and a size 12 for the short stem. The prostheses were then inserted until seated.

The distal 18 cm of each femur were sawn off, and the implanted proximal femurs were potted in polymethylmethacrylate (PMMA) bone cement (within a metal cylinder). The cement was fixed to the cylinder with three screws to prevent rotation and left for 30 min to cure.

**Fig. 3:**
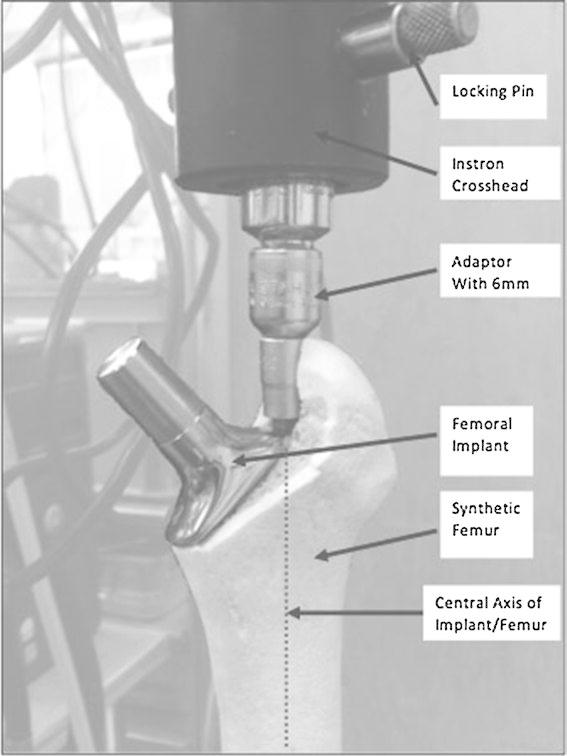
Image showing standardised set-up of equipment

The metal cylinder was mounted to the base of a servohydraulic testing machine (Instron 8874 Biaxial Testing System; Instron Corporation, MA, USA) using a bespoke adjustable vice. The potted bone was aligned such that the plane of the femoral stem was vertical, and directly underneath the centre of the servohydraulic crosshead. A 6-mm hex key was attached to the crosshead and lowered into the 6-mm hex hole in the implant (this hole is aligned with the centre of the distal femoral stem). This allowed the stem to be rotated about its central axis (Fig. 3).

Throughout the testing a small constant vertical load of 10 N was applied, to counteract any vertical loosening, and to ensure engagement of the hex key in the implant hex hole. Before each test, the Instron crosshead was manually positioned in a neutral position, fully engaged with the implant but with no vertical or rotational force.

To test resistance to femoral fracture, the implant was rotated clockwise through 90° in 1 s. This testing protocol has been described previously [13], and is designed to simulate peri-prosthetic fracture due to internal rotation on a planted foot, as might occur during stumbling.

Torque, rotation, vertical load and vertical position data were sampled 50 times per second throughout the testing protocols, and were exported to a data spreadsheet file (Microsoft Excel; Microsoft Corporation, WA, USA).

Following ethical approval, a single pair of cadaveric femurs were extracted from an embalmed cadaver donated to the Human Anatomy Unit (Charing Cross Hospital, London, UK). The cadaver had been embalmed with a mixture of formaldehyde, phenol, polyethylene glycol and alcohol, which has been shown not to significantly affect the stiffness of bone [14].

An experienced surgeon used a posterior approach and standard intra-operative techniques to implant and size the short and long femoral stems. The femurs were then carefully dissected from the cadavers and stripped of soft tissues.

The implanted femurs underwent the same experimental setup as the synthetic bones. Testing was in a clockwise direction on the left, and anticlockwise on the right femur to ensure both hips were torqued in internal rotation. The data was analysed using SPSS (IBM SPSS Statistics, version 20) using a Mann–Whitney *U* test as data was not found to be parametric.

## Results

The torsional force required to fracture the short-stem implanted femurs [mean 27.1 Nm, range 24.4–30.3, standard deviation (SD) 2.1] was significantly greater than that of the long stems (mean 24.2 Nm, range 21.1–30.1, SD 2.8) (Fig. 1; *p* = 0.03). The ranges of fracture torque for the short (24.4–30.3 Nm) and long (21.1–25.7 Nm) stems show only partial overlap, with the exception of a single outlier (30.1 Nm) in the long-stemmed group (excluding this value, the range was 21.05–25.70 Nm). The torsional force required to fracture the short-stem implanted cadaveric femur (27.8 Nm) was higher than that for the long stem (14.7 Nm).

**Fig. 4:**
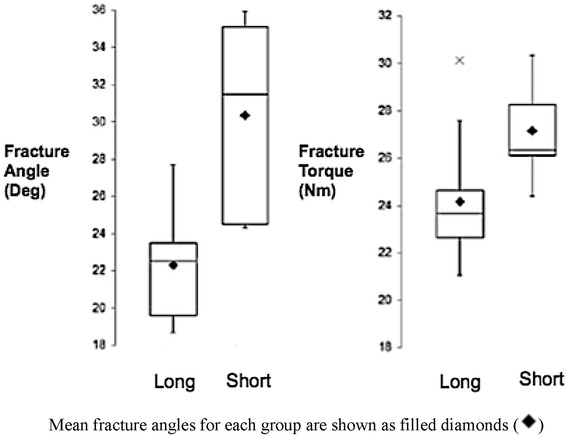
Box plots of the fracture angles and torque of long and short implanted stems

The angular deformation at fracture for the short stems (mean 30°, range 24°–36°, SD 5.2) was significantly greater than that of the long stems (mean 22^o^, range 19°–25°, SD 3.2, *p* = 0.002), (Fig. 1). The ranges of fracture angle for the short (24.3°–35.9°) and long (18.6°–27.7°) stems show only partial overlap. Fracture torque and angle data are presented in Fig. 4. The cadaveric bone fracture angle was 14.5° for the short stem, but was not clearly determinable for the longer stem.

**Fig. 5:**
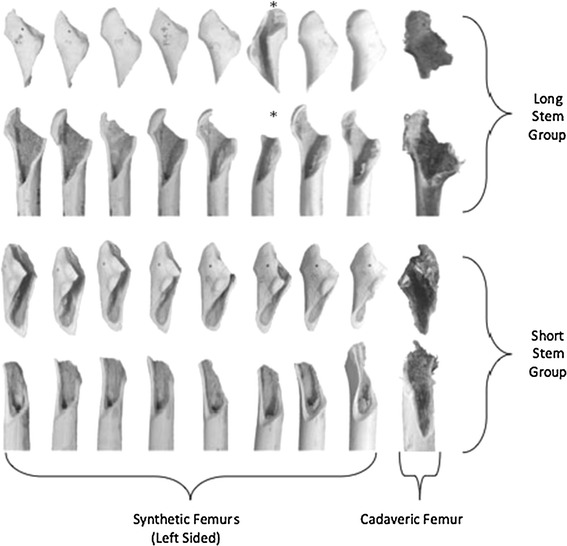
Photograph showing fracture patterns of the long- and short-stem groups

The fracture patterns for the two implants were consistent but different. Both stems displayed a spiral fracture pattern with the apex of fracture 3 cm below the lesser trochanter. However, the long-stem group had a butterfly segment of the anterior part of the greater trochanter but the short-stem group’s involved the entire greater trochanter (Fig. 5). The single outlier from the long-stem group (which fractured at 30.1 Nm) had a similar fracture pattern to the short stems.

## Discussion

In this study, we sought to compare the pattern and force required to induce a peri-prosthetic fracture of femurs implanted with uncemented short- and long-stem hip replacements. We found that bones implanted with the short-stemmed implants required a significantly higher force before fracture. Implanted femurs were also found to be more flexible and deformed more prior to fracture in the short-stem group. Although limited, testing in paired cadaveric femurs demonstrated a similar fracture torque and pattern to the results seen with synthetic bones. Our findings are consistent with a similar study [13] where the torsional fracture strength of *cemented* femoral components (in a synthetic bone model) demonstrated fracture torques of 25–40 Nm and fracture angles of 20°–35°. Jakubowitz et al. [10] compared the grit-blasted short uncemented Mayo^®^ hip (Zimmer, Warsaw, IN, USA) to an equivalent uncemented long-stem design. Whilst these implants differed from those in our study in many ways, the authors similarly found that the short-stem implants compared favourably to the long-stemmed equivalent with respect to the risk of a peri-prosthetic fracture.

As the short stem in our study is a relatively new addition to the implant market, we are not able to evaluate the fracture patterns we observed against clinical reports of peri-prosthetic fracture. However, given the clear and consistent difference between the fracture patterns of the two groups in synthetic and paired cadaveric femurs (Fig. 5), we can be confident that this difference is significant. Furthermore, Van Eynde et al. [15] have reported a typical fracture pattern in an uncemented long-stem series that was very similar to the fracture pattern we described for our long-stemmed implants.

The peri-prosthetic fracture pattern can have implications for recovery and treatment; however, as both fractures created an unstable femoral stem, revision of the stem would be necessary if they occurred in the early post-operative period [16].

Previous work by Cristofolini et al. [17] has demonstrated that the mechanical strength variability of cadaveric femurs may be up to 200 times that of composite synthetic femurs. These results help rationalise our choice in using mainly synthetic bones, which benefit from consistent geometries and mechanical properties. Their use also enabled accurate and reproducible implant positioning. In addition, previous studies have shown they do behave similarly to human femurs in mechanical testing protocols [13, 18–20]. Synthetic femurs may thus be a reasonable surrogate for human bone and our cadaveric testing results further support this conclusion.

A limitation of this in vitro study is that we are only able to simulate initial implant behaviour. The on-growth of bone onto the implanted femoral stems, promoted by the hydroxyapatite (HA) coating, only occurs in living bone. HA in living subjects would therefore increase the strength of implant fixation [21]. The present study is therefore most relevant in the context of implant behaviour in the early post-operative period, before full bony on-growth occurs.

We are also limited in our interpretation of the data by the fact that we only tested one size of each implant in a “medium”-sized synthetic femur. We cannot therefore comment on how implant sizing might affect biomechanical behaviour. Further work could investigate the effects of implant sizing on peri-prosthetic fracture risk.

In conclusion, we found that the peri-prosthetic fracture pattern of the two stems were different. In spite of this, both patterns would require stem revision and hence present a similar revision dilemma. However, the new short-stemmed press-fit femoral component allows more femoral flexibility and confers a higher resistance to peri-prosthetic fracture from torsional forces than the long stem. This higher resistance to fracture is an important consideration when selecting implants for elderly female patients who are both more likely to fall and to have osteoporotic bone.
